# Relationships Between Housing and Food Insecurity, Frequent Mental Distress, and Insufficient Sleep Among Adults in 12 US States, 2009

**DOI:** 10.5888/pcd11.130334

**Published:** 2014-03-13

**Authors:** Yong Liu, Rashid S. Njai, Kurt J. Greenlund, Daniel P. Chapman, Janet B. Croft

**Affiliations:** Author Affiliations: Rashid S. Njai, Kurt J. Greenlund, Daniel P. Chapman, Janet B. Croft, Centers for Disease Control and Prevention, Atlanta, Georgia.

## Abstract

**Introduction:**

Housing insecurity and food insecurity may be psychological stressors associated with insufficient sleep. Frequent mental distress may mediate the relationships between these variables. The objective of this study was to examine the relationships between housing insecurity and food insecurity, frequent mental distress, and insufficient sleep.

**Methods:**

We analyzed data from the 2009 Behavioral Risk Factor Surveillance System in 12 states. Housing insecurity and food insecurity were defined as being worried or stressed “sometimes,” “usually,” or “always” during the previous 12 months about having enough money to pay rent or mortgage or to buy nutritious meals.

**Results:**

Of 68,111 respondents, 26.4% reported frequent insufficient sleep, 28.5% reported housing insecurity, 19.3% reported food insecurity, and 10.8% reported frequent mental distress. The prevalence of frequent insufficient sleep was significantly greater among those who reported housing insecurity (37.7% vs 21.6%) or food insecurity (41.1% vs 22.9%) than among those who did not. The prevalence of frequent mental distress was also significantly greater among those reporting housing insecurity (20.1% vs 6.8%) and food insecurity (23.5% vs 7.7%) than those who did not. The association between housing insecurity or food insecurity and frequent insufficient sleep remained significant after adjustment for other sociodemographic variables and frequent mental distress.

**Conclusion:**

Sleep health and mental health are embedded in the social context. Research is needed to assess whether interventions that reduce housing insecurity and food insecurity will also improve sleep health and mental health.

## Introduction

Healthy People 2020 and the World Health Organization maintain that social determinants of health, including social, economic, and physical environments, shape people’s opportunities to achieve optimal health (1,2). Furthermore, the 5-tier health impact pyramid suggests that interventions related to the improvement of socioeconomic factors may have the greatest potential impact to change the social context to enable an individual’s default decisions to be healthy (3). Housing insecurity and food insecurity have been identified as 2 important social determinants of health (4,5). The definitions of housing insecurity vary and include having high housing costs relative to household income, living in environments of poor quality and unstable neighborhoods, living in overcrowded housing, or being homeless (6). In 2010, more than a quarter of US households (20.2 million households) paid more than 50% of their incomes for housing (7). Food insecurity can be defined as uncertainty about one’s ability to access safe and nutritious foods because of restricted financial resources (8). Nearly 15% of US households (approximately 48.8 million Americans) were food insecure at some time during 2010; 10.5% of US households were food insecure during 2000 (9). Research suggests that food insecurity is associated with the consumption of more high-energy–dense foods that may lead to weight gain, diabetes, and poor physical health (10,11). Studies also indicate that food insecurity is associated with psychological distress, anxiety, and depression among low-income women and children (12,13). Psychological distress may, in turn, adversely affect people’s physical health, mental health, and sleep (14).

Emerging data suggest that insufficient sleep is associated with obesity, diabetes, cardiovascular disease, stroke, high blood pressure, arthritis, asthma, anxiety, and depression (15–20); the rate of poor sleep is higher among women than men and is correlated with low household income, poverty, and unemployment (21–23). In 2008, 37.9% of US adults reported 14 days or more of insufficient sleep (24). Few studies have addressed the relationship between housing insecurity and insufficient sleep (21,22). The Behavioral Risk Factor Surveillance System (BRFSS), the largest telephone surveillance system in the world, recently began collecting data on insufficient sleep, defined as the number of days that the respondent felt that he or she did not get enough rest or sleep in the previous 30 days. The survey also began collection data on housing insecurity and food insecurity among US adult respondents (25). Frequent mental distress (≥14 days of mental distress during the previous 30 days) is a reliable indicator of psychological distress (26) and is also highly associated with insufficient sleep (18–22). The objective of this study was to assess the relationships between housing insecurity, food insecurity, frequent mental distress, and insufficient sleep.

## Methods

The BRFSS, a state-based, random-digit–dialed telephone survey of noninstitutionalized, civilian US adults aged 18 or older, consists of a core set of questions on public health issues that are asked in all states and optional modules that states may elect to include. In 2009, an optional module on social context, which included questions on housing insecurity and food insecurity, was administered in 12 states (Alabama, Arkansas, California, Hawaii, Illinois, Kansas, Louisiana, Nebraska, New Mexico, Oklahoma, South Carolina, and Wisconsin). Data from 68,111 (90.7%) of 75,103 respondents to this module were analyzed after excluding those who did not respond to questions about sleep (n = 1,320) and housing insecurity or food insecurity (n = 5,672). The response rate in these 12 states ranged from 42.8% to 66.9% (median, 58.2%) (27).

### Variables

The outcome variable, perceived insufficient sleep, was obtained from self-reported responses to the core question “During the past 30 days, for about how many days have you felt you did not get enough rest or sleep?” Frequent insufficient sleep, defined as not getting enough rest or sleep 14 days or more in the previous 30 days, was used in this analysis. Research has shown significant relationships between insufficient sleep, chronic diseases, obesity, and other risk factors when insufficient sleep was defined as 14 days or more in the previous 30 days without sufficient sleep (15–20).

Independent variables in this study were housing insecurity and food insecurity. The social context module included the questions, “How often in the past 12 months would you say you were worried or stressed about having enough money” to pay your rent or mortgage (housing insecurity) or to buy nutritious meals (food insecurity)? Response options to both questions were “never,” “rarely,” “sometimes,” “usually,” and “always.” We classified respondents as having housing insecurity or food insecurity if they reported that they “always,” or “usually,” or “sometimes” felt worried or stressed.

Covariates in this study were age (18–44 y, 45–64 y, ≥65 y), sex, race/ethnicity (non-Hispanic white, non-Hispanic black, Hispanic, and other non-Hispanic), and years of education (<12 y, 12 y or high school equivalency diploma, or >12 y). We examined a potential mediation variable, frequent mental distress, defined as the respondents reporting 14 days or more in the previous 30 days that they did not feel their mental health was good. Mental health is defined by the BRFSS question as including “stress, depression, and problems with emotions.” Frequent mental distress has been analyzed as an indicator of prolonged mental distress and is associated with insufficient sleep and sleep duration (18,20,28).

### Statistical analysis

First, we generated the distribution of the study population by the selected characteristics. Second, we conducted bivariate analyses of frequent insufficient sleep, frequent mental distress, housing insecurity, and food insecurity associated with the selected characteristics. Third, the adjusted prevalence ratios (PRs) and 95% confidence intervals (CIs) characterizing the relationships of frequent insufficient sleep with housing insecurity and food insecurity were obtained from separate multivariate logistic regression models after controlling for age, sex, race/ethnicity, and educational attainment. We conducted further analyses to examine whether frequent mental distress mediates these relationships. The magnitude and significance of the mediating effect was assessed by measuring the percentage change in the PR between a model with a specific mediator and a model without it [(PR_no mediator_ − PR_with a mediator_)/(PR_no mediator_ − 1)] × 100 (29,30). To be conservative, we noted a change of 20% or more in the PR (30) only when housing insecurity or food insecurity was significantly associated with both frequent mental distress and frequent insufficient sleep and frequent mental distress was also significantly associated with frequent insufficient sleep (31). We considered frequent mental distress to have a partial mediating effect on the relationship between housing or food insecurity and frequent insufficient sleep if the relationship remained significant after frequent mental distress was added to the model and a complete mediating effect if the relationship was no longer significant after frequent mental distress was added to the model (31). SAS-callable SUDAAN software was used to account for the complex sampling design (32). The significance level was denoted at *P* < .05.

## Results

Of the 68,111 survey respondents in this study, 50.8% were aged 45 or older, 62.2% were non-Hispanic white, 61.9% had more than 12 years education, 28.5% reported housing insecurity and 19.3% reported food insecurity during the previous 12 months, and 10.8% reported frequent mental distress and 26.4% reported frequent insufficient sleep during the previous 30 days (Table 1). In addition, a significantly higher percentage of non-Hispanic blacks, Hispanics, and other non-Hispanics than non-Hispanic whites reported food insecurity (*P* < .001) and housing insecurity (*P* < .001) (Figure). A significantly higher percentage of respondents who had 12 years or less of education reported food insecurity (*P* < .001) and housing insecurity (*P* < .001) than those with more than 12 years of education.

**Table 1:** Distribution of Selected Characteristics Among Adults 18 Years or Older in 12 US States,^a^ 2009

Characteristic	N^b^	% (95% Confidence Interval)^c^
**Total**	68,111	100.0
**Sex**		
Male	25,739	48.8 (47.8–49.7)
Female	42,372	51.2 (50.3–52.2)
**Age, y**		
18–44	17,309	49.2 (48.2–50.1)
45–64	29,203	34.4 (33.6–35.2)
≥65	21,259	16.4 (15.9–16.9)
**Race/ethnicity**		
Non-Hispanic white	48,802	62.2 (61.1–63.2)
Non-Hispanic black	7,265	9.2 (8.7–9.7)
Hispanic	4,477	19.2 (18.1–20.2)
Other	7,007	9.5 (8.9–10.1)
**Education, y**		
<12	6,377	11.6 (10.9–12.4)
12	20,257	26.5 (25.7–27.3)
>12	41,395	61.9 (60.9–62.8)
**Housing insecurity^d^**	14,334	28.5 (27.6–29.5)
**Food insecurity^e^**	12,166	19.3 (18.5–20.2)
**Frequent mental distress^f^**	6,965	10.8 (10.2–11.4)
**Frequent insufficient sleep^g^**	16,238	26.4 (25.6–27.2)

a Adult population was drawn from respondents to an optional module from the 2009 Behavioral Risk Factor Surveillance System in 12 states (Alabama, Arkansas, California, Hawaii, Illinois, Kansas, Louisiana, Nebraska, New Mexico, Oklahoma, South Carolina, and Wisconsin).

b Unweighted sample size.

c Weighted percentage and 95% confidence interval.

d Housing insecurity was defined as a response of “always,” “usually,” or “sometimes” felt worried or stressed about having enough money to pay rent or mortgage.

e Food insecurity was defined as a response of “always,” “usually,” or “sometimes” felt worried or stressed about having enough money to buy nutritious meals.

f Frequent mental distress was defined as a response of ≥14 days that mental health was not good in the past 30 days.

g Frequent insufficient sleep was defined as a response of ≥14 days of not getting enough rest/sleep in the past 30 days.

**Figure:**
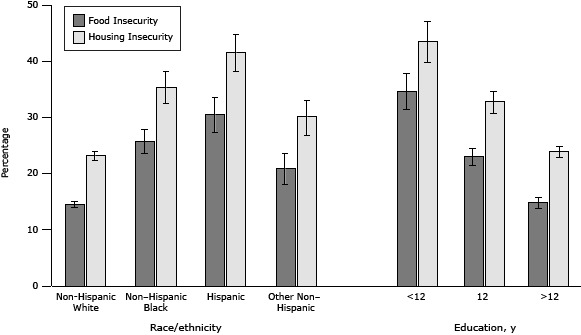
Percentage of housing insecurity and food insecurity by race/ethnicity and educational levels among adults aged 18 or older in 12 states, Behavioral Risk Factor Surveillance System, 2009. Error bars indicate 95% confidence intervals. **Table:** 

Characteristic	Food Insecurity, % (95% CI)	Housing Insecurity, % (95% CI)
**Race/ethnicity**		
Non-Hispanic white	14.6 (14.0-15.3)	23.2 (22.3–24.0)
Non–Hispanic black	25.8 (23.8–27.9)	35.3 (32.4–38.4)
Hispanic	30.5 (27.5–33.8)	41.5 (38.1–44.8)
Other non–Hispanic	20.9 (18.3–23.8)	30.1 (26.8–33.5)
**Education, y**		
<12	34.7 (31.5–38.0)	43.6 (39.9–47.3)
12	23.0 (21.4–24.6)	32.8 (30.9–34.8)
>12	14.9 (14.0–15.9)	23.9 (22.9–25.0)

Bivariate analyses (Table 2) showed that the prevalences for both frequent insufficient sleep and frequent mental distress were significantly higher among women than among men and among people younger than 65 than among those aged 65 or older (*P* < .001). A significantly greater percentage of non-Hispanic blacks reported frequent insufficient sleep (*P* = .02) and frequent mental distress (*P* < .001) than non-Hispanic whites. The percentage of Hispanics reporting frequent insufficient sleep was similar to that reported by non-Hispanic whites (*P* = .17), but a significantly greater percentage of Hispanics reported frequent mental distress (*P* < .001). The prevalence of frequent insufficient sleep did not differ significantly by years of education, but the prevalence of frequent mental distress was negatively associated with years of education (*P* < .001). The unadjusted prevalence of frequent insufficient sleep was significantly higher among respondents who reported either housing insecurity or food insecurity than among those who did not (*P* < .001). Respondents who reported either housing insecurity or food insecurity were about 3 times as likely to report frequent mental distress than were those who did not, and those who reported frequent mental distress were more than twice as likely to report frequent insufficient sleep as those who did not (59.7% vs 22.3%).

**Table 2:** Prevalence^a^ of Frequent Insufficient Sleep^b^ and Frequent Mental Distress^c^ Among Adults 18 Years or Older in 12 US States,^d^ 2009

Characteristic	Frequent Insufficient Sleep, % (95% CI)	*P* Value^e^	Frequent Mental Distress, % (95% CI)	*P* Value^e^
**Sex**				
Men	23.9 (22.6–25.2)	1 [Reference]	8.8 (7.9–9.7)	1 [Reference]
Women	28.8 (27.8–29.8)	<.001	12.6 (11.8–13.4)	<.001
**Age, y**				
18–44	30.7 (29.3–32.2)	<.001	11.1 (10.1–12.1)	<.001
45–64	26.1 (25.0–27.3)	<.001	12.0 (11.1–12.9)	<.001
≥65	14.1 (13.1–15.1)	1 [Reference]	7.3 (6.3–8.2)	1 [Reference]
**Race/ethnicity**				
Non-Hispanic white	26.5 (25.6–27.3)	1 [Reference]	9.6 (9.0–10.1)	1 [Reference]
Non-Hispanic black	29.4 (27.0–31.9)	.02	14.8 (12.5–17.1)	<.001
Hispanic	24.5 (21.8–27.1)	.17	13.5 (11.3–15.6)	<.001
Other	27.1 (24.0–30.2)	.69	9.2 (7.5–11.0)	.71
**Education, y**				
<12	26.1 (23.2–28.9)	.84	16.3 (14.0–18.6)	<.001
12	26.6 (25.0–28.2)	.81	12.6 (11.3–14.0)	<.001
>12	26.4 (25.4–27.4)	1 [Reference]	9.0 (8.3–9.6)	1 [Reference]
**Housing insecurity^f^**				
No	21.6 (20.7–22.5)	1 [Reference]	6.8 (6.2–7.4)	1 [Reference]
Yes	37.7 (35.7–39.6)	<.001	20.1 (18.5–21.7)	<.001
**Food insecurity^g^**				
No	22.9 (22.1–23.7)	1 [Reference]	7.7 (7.1–8.3)	1 [Reference]
Yes	41.1 (38.7–43.4)	<.001	23.5 (21.7–25.3)	<.001
**Frequent mental distress (≥14 d/30 d)**				
No	22.3 (21.5–23.1)	1 [Reference]	—	
Yes	59.7 (56.7–62.7)	<.001	—	
**Frequent insufficient sleep (≥14 d/30 d)**				
No	—		5.9 (5.3–6.5)	1 [Reference]
Yes	—		24.4 (22.9–26.0)	<.001

Abbreviations: CI, confidence interval; —, does not apply.

a Prevalence (%) and 95% CI were weighted to take study design into account.

b Frequent insufficient sleep was defined as a response of ≥14 days of not getting enough rest/sleep in the past 30 days.

c Frequent mental distress was defined as a response of ≥14 days that mental health was not good in the past 30 days.

d Adult population was drawn from respondents to an optional module from the 2009 Behavioral Risk Factor Surveillance System in 12 states (Alabama, Arkansas, California, Hawaii, Illinois, Kansas, Louisiana, Nebraska, New Mexico, Oklahoma, South Carolina, and Wisconsin).

e Obtained from a 2-sided *t* test to assess the difference of prevalence of frequent insufficient sleep and frequent mental distress among groups.

f Housing insecurity was defined as a response of “always,” “usually,” or “sometimes” felt worried or stressed about having enough money to pay rent or mortgage.

g Food insecurity was defined as a response of “always,” “usually,” or “sometimes” felt worried or stressed about having enough money to buy nutritious meals.

Unadjusted results showed that respondents who reported housing insecurity or food insecurity were each more than 70% more likely to report frequent insufficient sleep than those who did not report the insecurity (Table 3, Model 1). Housing insecurity and food insecurity remained significantly associated with frequent insufficient sleep after adjustment for sociodemographic covariates for housing insecurity (PR = 1.67; 95% CI, 1.55–1.79) and for food insecurity (PR = 1.75; 95% CI, 1.62–1.88) (Table 3, Model 2). Although further adjustment for respondents’ frequent mental distress status reduced the magnitude of the relationship by 27% (Table3, Model 3), both housing insecurity and food insecurity remained significantly associated with frequent insufficient sleep, suggesting that frequent mental distress was only a partial mediator of the relationships of these social context variables with insufficient sleep.

**Table 3:** Prevalence Ratio and 95% Confidence Interval of Frequent Insufficient Sleep, by Housing Insecurity and Food Insecurity Among Adults 18 Years or Older in 12 US States,^a^ 2009

Characteristic	Model 1^b^	Model 2^c^	Model 3^d^
**Housing insecurity**			
No	1 [Reference]	1 [Reference]	1 [Reference]
Yes	1.74 (1.63–1.86)	1.67 (1.55–1.79)	1.49 (1.38–1.60)^e^
**Food insecurity**			
No	1 [Reference]	1 [Reference]	1 [Reference]
Yes	1.79 (1.68–1.92)	1.75 (1.62–1.88)	1.54 (1.42–1.67)^e^

a Adults were drawn from respondents to an optional module from the 2009 Behavioral Risk Factor Surveillance System in 12 states (Alabama, Arkansas, California, Hawaii, Illinois, Kansas, Louisiana, Nebraska, New Mexico, Oklahoma, South Carolina, and Wisconsin).

b Model 1: obtained from separate univariate logistic regression models that included only insecurity variables.

c Model 2: results adjusted for age, sex, race/ethnicity, and education.

d Model 3: results adjusted for all covariates in model 2 and for frequent mental distress.

e 20% to less than 40% reduction of prevalence ratio between model with and without the potential mediator.

## Discussion

Our results demonstrated that both housing insecurity and food insecurity were associated with frequent insufficient sleep among US adults in 12 states. This positive relationship was modestly attenuated but not completely explained by frequent mental distress. Although the mechanisms underlying the association are not clear, one potential explanation is that stress caused by housing insecurity or food insecurity could lead to prolonged psychological distress or depressive symptoms (13,33–35). Previous studies have also shown that housing insecurity, particularly crowding and multiple relocations, were associated with psychological distress, poor health, and developmental risk among children (33,34), and food insecurity was highly associated with mental distress among women (13,35) and school-aged children (12) even after adjustments for sociodemographic characteristics. In addition, poor housing conditions may also be related to poor sleep through concerns about personal safety, exposure to higher noise levels, and inadequate heating or cooling (36,37).

Other studies reported that low levels of food security were significantly associated with short sleep duration and suggested that the significant relationship might be due to racial/ethnic minority status and low socioeconomic position (21,22), findings that are also consistent with ours. Furthermore, hunger may acutely affect the ability to sleep well. Educational and racial/ethnic disparities in these social contexts may also play an important role in perceived insufficient sleep and frequent mental distress.

Chronic stress or worry related to poor housing and poor nutrition may interact with endocrine systems such as the sympathoadrenal medullary and the hypothalamo–pituitary–adrenocortical systems and are associated with elevated levels of cortisol, adrenocorticotropic hormone (ACTH), and corticotropin-releasing hormone (CRH). Research indicated that an elevated level of cortisol was highly associated with frequent microarousals (38). In addition, elevated ACTH levels could increase the frequency of morning awakening (39). Furthermore, the stages of slow-wave sleep and rapid eye movements in the sleep cycle could be reduced by excessive release of CRH (40). These physiological changes may cause sleep disturbances with increased cognitive arousal or cortical arousal or both (41).

Our results were consistent with previous studies in which either “housing instability” (definitions of which have included frequent moves, difficulty paying rent, spending more than half of household income on housing, being evicted, or living in overcrowded conditions) or “food insufficiency” (respondents reported that their family sometimes or often did not get enough food to eat) was used to assess the relationship with mental health (12,13,33). As in these earlier cross-sectional studies, which examined the relationship of each social context with frequent insufficient sleep, we cannot assess causation.

Although this is the largest study to date to address the relationship between housing insecurity and food insecurity and insufficient sleep among US adults, it has several limitations. First, because it was a cross-sectional study, we cannot establish causation. Second, because our results are based on the analysis of information provided by BRFSS in only 12 states and BRFSS does not include people residing in institutions such as nursing homes and prisons, our results are not generalizable to the entire US adult population. Third, the dichotomous measures of housing insecurity and food insecurity used in this study were each derived from responses to a single survey question on respondents’ confidence in their financial capacity to acquire adequate housing and food and therefore did not reflect all respects of housing insecurity and food insecurity. For example, the measure of housing insecurity did not address whether respondents resided in housing of poor quality, in overcrowded housing, or in unstable neighborhoods (6), and the measure of food insecurity did not reflect the extent to which respondents experienced hunger (9). However, the influence of the different definitions of housing insecurity and food insecurity on our results may be limited given the findings from previous studies that are consistent with ours (9,33,34). In addition, frequent insufficient sleep is a subjective measure of sleep health and was not corroborated by polysomnography. However, frequent insufficient sleep may partly reflect some sleep problems such as obstructive sleep apnea and is highly related to sleep duration (20). Efforts are needed to address the mediating role of other social determinants such as marriage, income, employment, accessibility of health services, and geographic variation in social context in the relationship of housing insecurity and food insecurity with poor sleep (22,23,42). Finally, our results could have been influenced by residual variations because other cofounders were not taken into account.

Fundamental societal transformations may be required to achieve social and economic changes that affect health (3). Potential improvements in housing and access to healthy food include community-level projects to provide environmentally healthful and safe housing for low-income families, food subsidy programs, and educational technology programs to enable low-income individuals to re-enter the workforce in new careers.

In summary, our findings, which are based on general-population data, add to a growing body of evidence that shows housing insecurity and food insecurity are associated with psychological distress and insufficient sleep. Our findings are consistent with previous findings showing that stress related to housing insecurity and food insecurity may sustain and adversely affect sleep and precipitate long-term adverse consequences to physical and mental health. Furthermore, our findings support the idea that sleep health and mental health are embedded in the social context. Research is needed to assess whether interventions that reduce housing insecurity and food insecurity will also improve those outcomes.
